# Validation of physical activity recall questionnaire and log using doubly-labelled water in Sri Lankan children

**DOI:** 10.1038/s41430-025-01579-0

**Published:** 2025-02-18

**Authors:** Prasangi Dabare, Pujitha Wickramasinghe, Indu Waidyatilaka, Sarita Devi, Maduka de Lanerolle Dias, Rajitha Wickremasinghe, Dulani Samaranayake, Ranil Jayawardena, Andrew P. Hills, Pulani Lanerolle

**Affiliations:** 1 Department of Physiotherapy, Faculty of Allied Health Sciences, General Sir John Kotelawala Defense University, Colombo, Sri Lanka; 2 Department of Pediatrics, Faculty of Medicine, University of Colombo, Colombo, Sri Lanka; 3 Department of Biochemistry and Molecular Biology, Faculty of Medicine, University of Colombo, Colombo, Sri Lanka; 4 St. John’s Research Institute, St. John’s National Academy of Health Sciences, Bangalore, Karnataka India; 5 Department of Public Health, Faculty of Medicine, University of Kelaniya, Ragama, Sri Lanka; 6 Department of Community Medicine, Faculty of Medicine, University of Colombo, Colombo, Sri Lanka; 7 Department of Physiology, Faculty of Medicine, University of Colombo, Colombo, Sri Lanka; 8 School of Health Sciences, College of Health and Medicine, University of Tasmania, Hobart, Australia

**Keywords:** Health care, Medical research

## Abstract

**Background and objective:**

Valid and cost-effective approaches are essential to assess physical activity and sedentary behaviours in individuals of all ages. This study aimed to validate a physical activity recall questionnaire (PAR-Q) and a physical activity log against the doubly labelled water (DLW) technique in 11-13-year-old Sri Lankan children.

**Method:**

Total energy expenditure (TEE) was determined using the criterion DLW technique (TEE_DLW_) and physical activity energy expenditure (PAEE_DLW_) was estimated. Subsequently, physical activity assessment methods were validated in a group of children (*n* = 79). PAR-Q was adapted from the adolescent physical activity questionnaire and the physical activity log from the Bouchard activity diary. The youth physical activity compendium was used to calculate PAEE from both PAR-Q (PAEE_PAR-Q_) and activity log (PAEE_PALog_). Statistical analysis included Pearson’s correlation coefficient and Bland-Altman analyses.

**Results:**

Mean age of the study sample was 12.0 ± 0.8 years where the greater proportion were males (51.9%, *n* = 41). PAEE_PAR-Q_ and PAEE_PALog_ were significantly correlated with the PAEE_DLW_ (*r* = 0.69 and *r* = 0.49, *p* < 0.05). PAR-Q underestimated PAEE by 230.1 ± 1071.1 kJ/day. Physical activity log overestimated PAEE by 14.6 ± 1283.2 kJ/day; for unit increase in the mean of the two measurements, the difference between the measurements increased by 0.48 (R^2^ = 0.16, *p* < 0.001).

**Conclusion:**

PAR-Q is a valid tool for predicting PAEE in Sri Lankan children whereas the physical activity log was not. Researchers and practitioners can confidently use the PAR-Q to assess physical activity in this population, with potential applications in various research and intervention settings.

## Introduction

Valid and reliable physical activity assessment methods are a prerequisite to monitor physical activity levels and assess the effectiveness of interventions in any population [1]. The accurate assessment of physical activity level, including intensity, duration, and PAEE, is challenging and requires reliable assessment methods [2], however a wide range of subjective and objective measures are available. Among these methods, interviewer-administered and self-administered questionnaires, proxy reports from parents and teachers, and physical activity diaries/logs have been used to estimate physical activity levels in children and adolescents. The attractiveness of physical activity questionnaires and physical activity logs/diaries is their suitability in studies with a large number of individuals as they are cost-effective, easy to administer and practical [3]. Commonly, interviewer-administered questionnaires are more effective in gathering information related to physical activity compared to self-administered questionnaires [4].

To date, there are no published data on validation of physical activity questionnaire or activity log for 11-13-year-old children in Sri Lanka. Additionally, the globally available subjective measures of physical activity are only designed to assess the physical activity individuals perform during school time. Further, such approaches do not provide estimates of energy expenditure and details on the frequency, time, and intensity of different physical activities. Due to the subjective nature of physical activity questionnaires and activity logs, it is crucial to validate them against the criterion doubly labelled water (DLW) technique [5] before being used in a specific population [4]. Although the relative cost of the DLW technique is high, which typically precludes its use in large samples, its high accuracy allows the validation of other field methods [5]. This study aimed to validate physical activity by the PAR-Q and activity log, which capture type, frequency, intensity, duration and PAEE, against the DLW technique among Sri Lankan children aged 11–13 years.

## Methods

### Study participants, selection and study design

A purposive sample of 96 children aged 11–13 years were recruited from the Colombo Municipal Council area. The study was designed and conducted according to the Helsinki Declaration. Informed written consent from parents and assent from children were obtained. The Ethics Review Committee of the Faculty of Medicine, University of Colombo, Sri Lanka (EC/16/192) approved the study. A total of 16 children free of any acute or chronic medical conditions or prolonged use of medication, were recruited from each age group (11, 12 and 13 years) according to the national distribution of nutritional status. Details of the study design and method of participant selection and recruitment are published elsewhere [6, 7].

### Measurements

#### Anthropometric measurements

Height and weight were measured according to the International Society for the Advancement of Kinanthropometry protocol [8] using a stadiometer (Seca 225 by SECA GmbH & Co. Kg., Hamburg, Germany) and a calibrated electronic scale (Seca 803 by SECA GmbH & Co. Kg., Hamburg, Germany), respectively. BMI was calculated as weight divided by height squared (kg/m^2^).

#### DLW technique for TEE calculation

The two-point DLW protocol was used according to the International Atomic Energy Agency protocol (IAEA) [5]. A weighted mixture of 0.12 g.kg^−1^ body water of 99.8% ^2^H_2_O and 1.8 g.kg^−1^ body water of 10% H_2_^18^O (Sigma-Aldrich Co., St. Louis, MO, USA) was used as the DLW dose [5]. Prior to administering the DLW dose on the dosing day (Day 1), a baseline urine sample was taken. The first post-dose urine sample was taken four hours after the dose was administered. The last urine sample was taken on day 10 at the same time as the first urine sample after the dosing, which was taken four hours after the dose on day 1. Before analysis, all samples were kept in storage at -20°C. Further, details of the DLW procedure, including dose preparation, dosing, sample collection, sample storage, analysis and calculation of energy expenditure are published elsewhere [6, 7].

Urine samples were analyzed at the Mass Spectrometry Laboratory, St. John’s Research Institute, Bangalore, India using isotope-ratio mass spectrometry (IRMS, Delta V Advantage, Thermo Scientific, Bremen, Germany). Total body water (TBW) was estimated and lean body mass (FFM) derived from the TBW value. According to the two-compartment model of body composition assessment, the body weight is comprised of FM and FFM. FFM was estimated from the corrected TBW using the hydration coefficient [5]. The hydration coefficient among the children was calculated using the age-specific hydration constants [9]. Assuming a 2-compartment model, FFM was subtracted from total body weight to calculate fat mass (FM). The rate of carbon dioxide production was calculated by the difference in the ^2^H and ^18^O turn-over rates using the equation of Schoeller et al. [10]. This was corrected for the non-aqueous isotope exchange and isotope fractionation. Total energy expenditure (TEE_DLW_) was calculated using the modified Weir equation [11], and basal metabolic rate (BMR) was calculated using the Schofield et al. [12] equation. Assuming 10% of TEE is allocated to the thermic effect of food [13], PAEE (PAEE_DLW_) was calculated according to the standard criteria of IAEA Human Health Series 3 [5].

### PAR-Q

The physical activity questionnaire used in the current study was an interviewer-administered instrument adapted from the adolescent physical activity recall questionnaire (APAR-Q) [14]. As in the APAR-Q, the current PAR-Q included questions regarding organized/structured physical activities (i.e., sports, games, exercise programmes) and other non-organized physical activities (leisure time/household activities). In addition, to enhance the recall ability, each weekday was divided as: before, during and after school time and questions were included to collect activities performed at different intensities. In each category, questions were included about the activity type, frequency of each activity performed and the time spent on each occasion. The final PAR-Q consisted of 3 sections: organized/structured physical activities (i.e., sports, games, exercise programmes), other non-organized physical activities (leisure time/household activities), and sedentary behaviour (activities done while sitting such as studying, reading, watching television, playing video/computer games) performed during the past 7 days (weekdays and weekend days separately). After the initial design of the questionnaire, physical activity experts, sports medicine consultants, paediatricians, and physical education teachers provided feedback regarding wording and content. The questionnaire was then pretested with 20 adolescents aged between 11-13 years outside the defined study area. They were requested to provide comments on the clarity of the questions and any ambiguous areas. None of the students reported difficulty in understanding the questions and instructions provided and, hence, no amendments were required. The PAR-Q questionnaire was administered to all participants by the same investigator on day 7 following the DLW dosing and following a comprehensive explanation.

PAEE from the questionnaire (PAEE_PAR-Q_) was estimated using the method described by Butte et al. [15]. First, total minutes per week was calculated for each activity (minutes/week) and this was multiplied by the corresponding metabolic equivalent value (MET) for the activity from the Youth Compendium of Physical Activities (YCPA) to calculate the MET minutes/week per activity [15]. Then using predicted BMR [12], energy cost (kJ/day) was calculated.

### Physical activity log

The physical activity log was adapted from the Bouchard activity diary [16]. Physical activity data were recorded on three days (two weekdays and one weekend day) with each hour divided into 15-minute time periods. Experts including sports medicine consultants, paediatricians, and physical education teachers scrutinized the wording and the content of the log and it was pretested before administering to the study participants on Day 1 of the study. Both children and their parents were instructed on how to complete and monitor the log. Participants were requested to complete the log on any two ‘regular’ weekdays and one ‘regular’ weekend day within the DLW study period [16] and to record activities over the course of the day as they occurred. They were specifically advised to select typical days that reflected their normal routine activities. Completed activity logs were collected on the last post-dose urine sample collection day (Day 10). Energy expenditure from the physical activity log (PAEE_PALog_) was estimated by the same method as described above to calculate the PAEE_PAR-Q._

### Statistical analyses

Of the 96 children, four were eliminated as their post-dose urine sample enrichments, as determined by IRMS, were lower than the baseline enrichment for ^2^H and ^18^O. Five participants were excluded as the urine sample collection for the DLW protocol was not completed. Eight more participants’ findings were eliminated from the study due to the identification of those values as outliers (>±3 standard deviations from the mean in each data column). Hence, the final sample consisted of 79 children. The information regarding the data-cleaning procedure is published elsewhere [7]. Data analysis was carried out using the SPSS statistics software (version 23.0), and the Kolmogorov-Smirnov test was used to assess the normality of data. Data are presented as mean and standard deviation (SD).

Pearson’s correlation coefficient was used to assess the association between energy expenditure values obtained from the criterion method (TEE_DLW_ and PAEE_DLW_) and physical activity values obtained from the PAR-Q and activity log (PAEE_PAR-Q_ and PAEE_PALOG_). Paired sample *t*-test was used to compare the energy expenditure values obtained from the criterion method with questionnaire and activity log. The Bland–Altman technique of assessing agreement between methods [17] was used to assess the agreement of PAEE estimated from the criterion method with the PAEE from the questionnaire and activity log. In this method, differences between the PAEE_DLW_ and PAEE_PAR-Q_ or PAEE_PALOG_ (y-axis) were plotted against the average of PAEE_DLW_ and PAEE_PAR-Q_ or PAEE_PALOG_ (x-axis). Upper and lower 95% agreement limits were calculated as mean bias ± 1.96 x standard deviations [17]. The relationship between the differences of the measurements and the average of the measurements was evaluated by simple linear regression analyses.

## Results

The mean age of the participants was 12.0 (±0.8) years and 52% of the sample comprised males (*n* = 41). The mean weight, height, FM and FFM of the sample were 35.2 (±7.7) kg, 1.5 (±0.1) m, 10.1 (±4.9) kg and 25.1 (±4.6) kg, respectively.

Energy expenditure values of the study population are shown in Table 1.

**Table 1:** Energy expenditure values by DLW, PAR-Q and physical activity log.

Characteristic	Total *(n* = *79)*
	Mean ± SD
TEE_DLW_ (kJ/day)	7942.1 ± 1816.3
PAEE_DLW_ (kJ/day)	2148.1 ± 1440.6
PAEE_PAR-Q_ (kJ/day)	1918.0 ± 1215.9
PAEE_PALog_ (kJ/day)	2162.7 ± 951.0

*TEE* total energy expenditure, *PAEE* physical activity energy expenditure, *DLW* doubly labelled water, *PAR-Q* physical activity recall questionnaire, *PALog* physical activity log (1 kcal = 4.186 kJ).

**Fig. 1:**
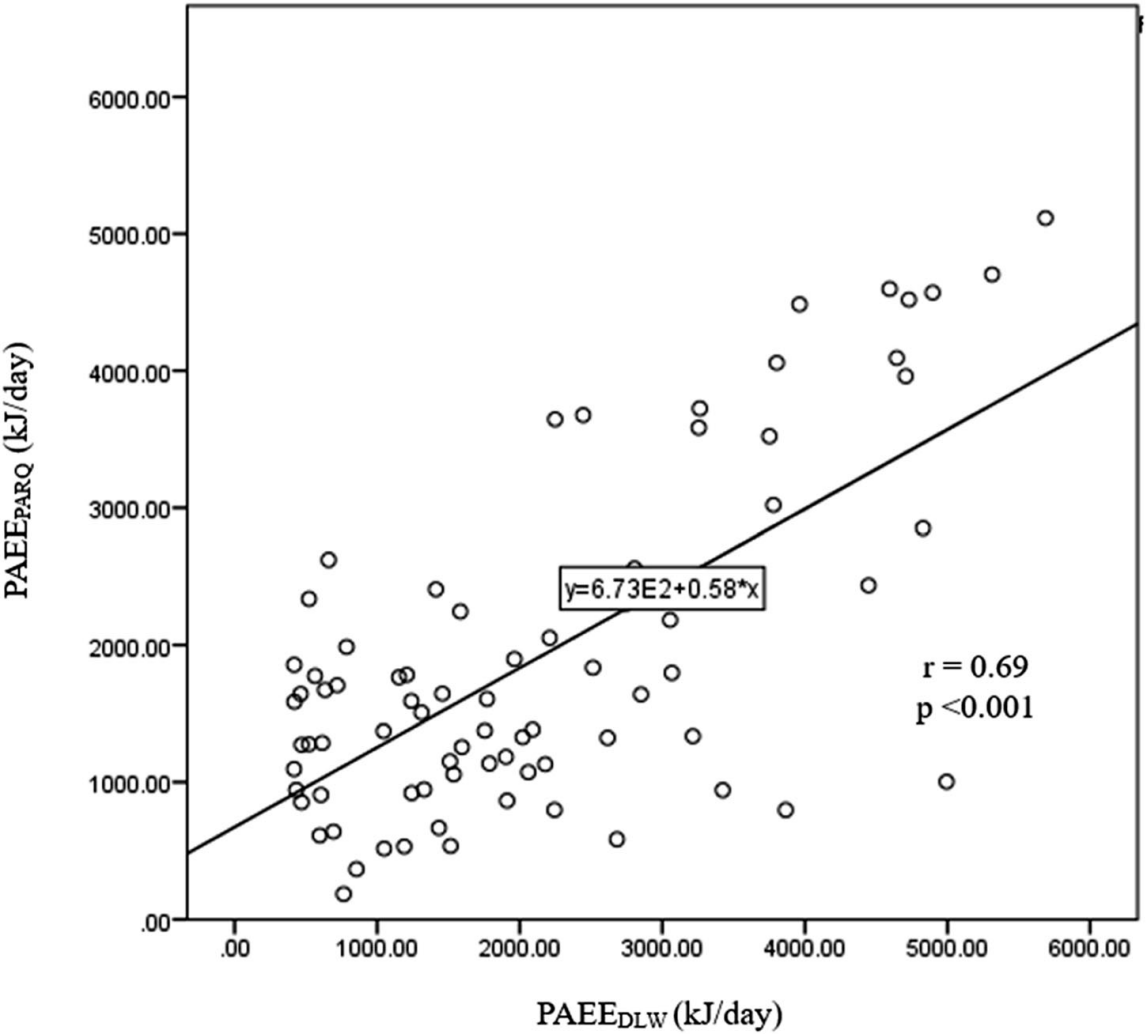
Regression line of PAEE calculated using the PAR-Q *vs*. DLW technique.

PAEE_PAR-Q_ was significantly correlated with the PAEE_DLW_ in the total sample (*r* = 0.69, *p* < 0.05) (Fig. 1). On average, PAR-Q underestimated PAEE by 230.1 kJ/day.

There was no significant (*p* = 0.06) difference between the PAEE calculated by the DLW technique and PAEE estimated by the questionnaire (Table 2).

**Table 2:** Comparison of PAEE by PAR-Q and physical activity log with criterion DLW.

Energy expenditure methods	Mean difference^a^ ± SD (kJ/day)	*P* value
PAEE_DLW_ *vs*. PAEE_PAR-Q_	230.1 ± 1071.1	0.06
PAEE_DLW_ *vs*. PAEE_PALog_	14.7 ± 1283.4	0.92

**Fig. 2:**
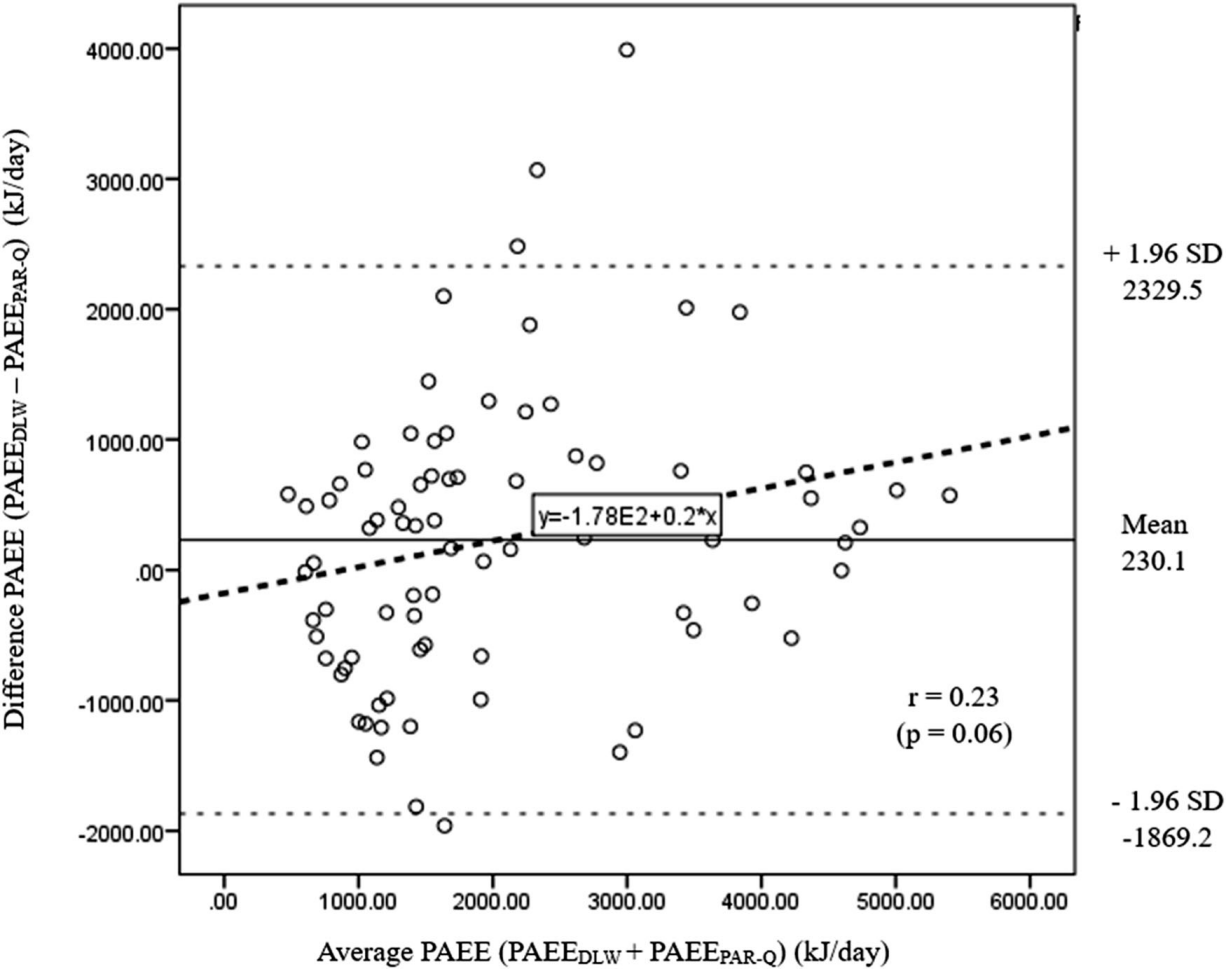
Differences between PAEE_DLW_ and PAEE_PAR-Q_ plotted against the average of PAEE_DLW_ and PAEE_PAR-Q_. Solid line indicates the mean bias and the two dashed lines the 95% upper and lower agreement limits (mean bias ± 1.96 x standard deviations). The thick dashed line indicates the regression line.

The limits of agreement between the PAEE estimated from the DLW technique with the PAEE calculated from the PAR-Q were assessed using the Bland–Altman analysis; a wide limit of agreement was observed with PAEE estimated using the PAR-Q (Fig. 2).

**Fig. 3:**
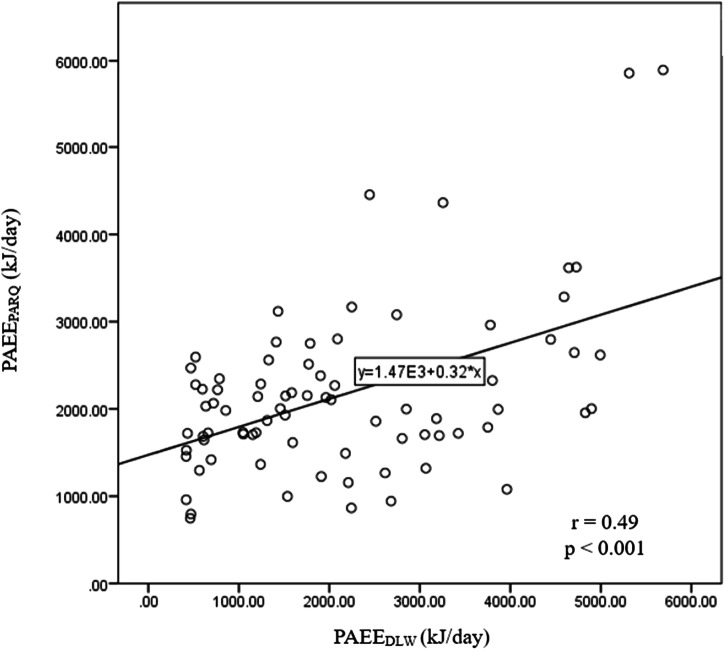
Regression line of PAEE calculated using the physical activity log *vs*. DLW technique.

PAEE calculated by physical activity Log (PAEE_PALog_) significantly correlated with the PAEE_DLW_ (*r* = 0.49, *p* < 0.05) in the total population (Fig. 3).

There was no significant difference between the PAEE calculated by the physical activity log and the criterion method (*p* = 0.92) (Table 2). On average, the physical activity log overestimated PAEE by 14.7 J/day among the total sample.

**Fig. 4:**
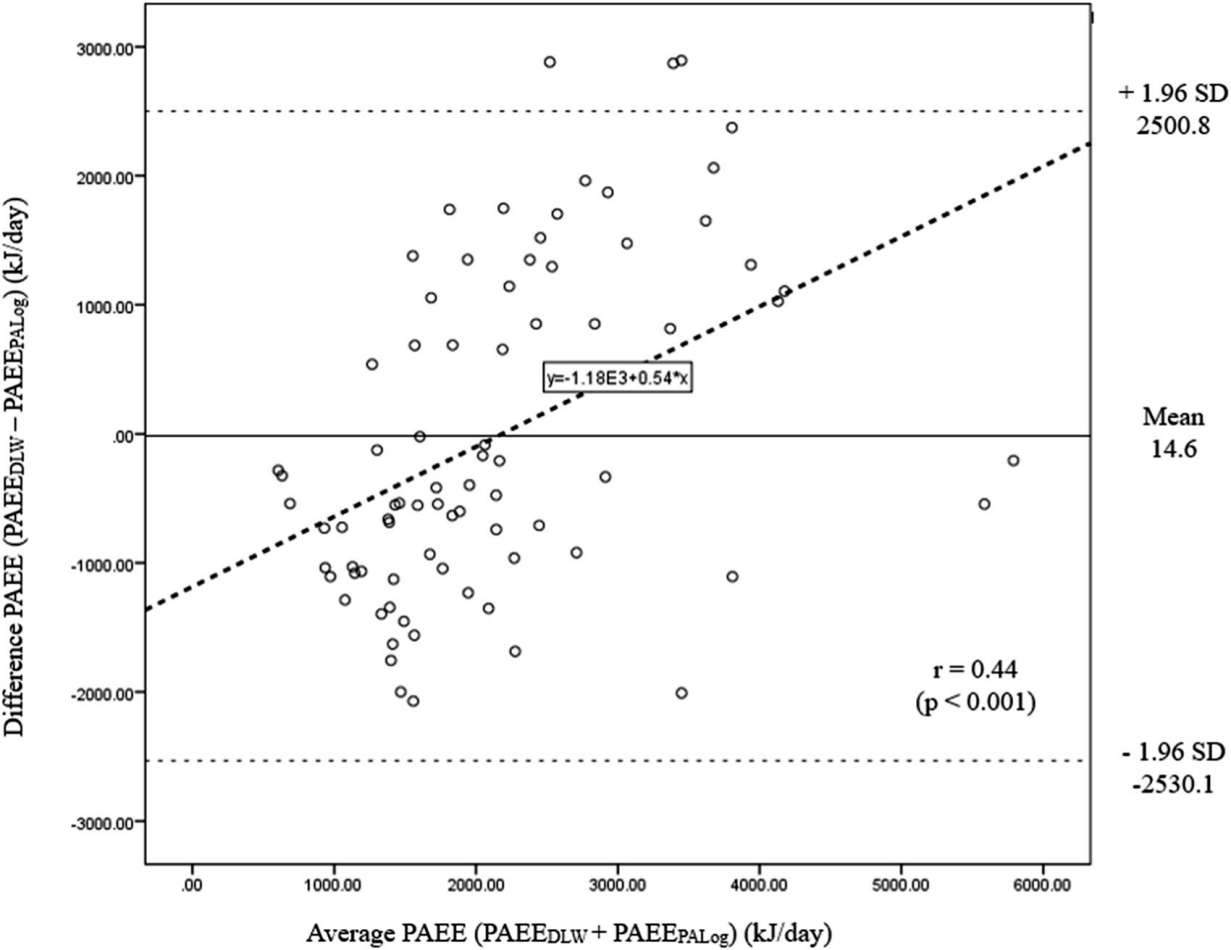
Differences between PAEE_DLW_ and PAEE_PALog_ plotted against the average of PAEE_DLW_ and PAEE_PALog_. Solid line indicates the mean bias and the two dashed lines indicate the 95% upper and lower agreement limits (mean bias ± 1.96 x standard deviations). The thick dashed line indicates the regression line.

The Bland-Altman plot displaying the degree of agreement between PAEE_DLW_ and PAEE_PALog_ is shown in Fig. 4; for each unit increase in the mean of the two measurements, the difference between the measurements increased by 0.48 (R^2^ = 0.16, *p* < 0.001). A significant (*p* < 0.05) correlation was observed between the average and difference of PAEE_DLW_ and PAEE_PALog_. As the PAFE measured by DLW increased, the overestimation of PAEE by physical activity log increased.

The average absolute difference compared to the DLW technique was found to be 842.7 kJ/day for the PAR-Q and 1099.8 kJ/day for the physical activity log. These results indicate a substantial average difference for both PAR-Q and the activity log when compared to DLW. The percent difference with the DLW technique was 41.5% for the PAR-Q and 51.0% for the physical activity log.

## Discussion

Recognizing the impact of steadily declining physical activity levels as a major risk factor for non-communicable diseases, and the need to improve risk profiles including physical inactivity and sedentary behaviour among children and youth, this study aimed to add value to currently available physical activity assessment tools for children and adolescents. To the best of our knowledge, this is the first study to have generated TEE data using the gold standard DLW technique and validate physical activity assessment approaches (PAR-Q and physical activity log) in Sri Lankan adolescents aged 11–13 years. Further, this is the first study to generate valid field tools to assess physical activity including type, frequency, intensity and duration.

In the current study, PAFE calculated from the criterion method had a correlation of 0.69 with PAEE calculated from the PAR-Q and a correlation of 0.49 with PAFE calculated using the physical activity log. The correlation between the PAFE’s calculated by the criterion method and the PAR-Q was higher than that reported in similar validation studies. Many studies have reported a low to moderate agreement between the physical activity assessment methods against criterion reference methods with the correlation coefficient ranging from 0.20 to 0.6 [18–23]. However, the criterion reference method used in these studies differed in each assessment; most had used accelerometers [1, 19–21]. Very few studies have used the DLW technique as the criterion [17, 22].

Most validation studies among children and adolescents reported a low to moderate agreement between physical activity assessment methods and the criterion reference as physical activity questionnaires and logs are primarily designed to assess physical activity and not PAEE. Hence, it is likely that all key activities contributing to daily energy expenditure are not accounted for, thereby reducing validity [23, 24]. According to Kohl and co-workers [19], physical activity assessment methods with shorter recall periods are more efficient than those with longer time periods. However, in contrast, the physical activity log used in the current study reported a poor correlation with the criterion method compared to that of the PAR-Q and a systematic bias in predicting energy expenditure. Though the physical activity log recorded activities in shorter time periods, these only accounted for three random days within the ten days of the DLW assessment period. Hence, it may not accurately reflect normal free-living daily physical activities and may have led to poor correlation with the PAEE estimated using the criterion technique.

In the current study, Bland–Altman plots were used to assess agreement between methods [17]. Using this approach, a wide limit of agreement with PAEE estimated using the PAR-Q and activity log suggested that both tools are not suitable to estimate PAEE. Additionally, the Bland–Altman plot showed a significant correlation between the average of the two measurements and the difference of PAEE estimated using DLW and activity log; PAFE measured by the activity log overestimated that of the criterion method when the average of the measurements increased. Overall, these findings underscore the importance of carefully selecting and validating appropriate methods for assessing physical activity and energy expenditure, particularly when precise individual-level estimations are required. Researchers should be cautious about the limitations and potential biases associated with each method when interpreting results in future studies.

The PAR-Q used in the current study was valid in estimating PAEE at a group level but underestimated PAEE in the total sample. Similar validation studies have also reported an underestimation of energy expenditure by self-reported and interviewer-administered physical activity assessment tools compared to the DLW or other criterion methods [18, 22–24]. Children have the ability to recall vigorous intensity activities more accurately compared to light intensity activities [25–27] but such physical activity also has a significant effect on the PAEE of individuals [28]. Hence, underreporting light intensity physical activity may have caused underestimation of PAEE by the PAR-Q.

The current study used the relatively new YCPA developed by Butte and colleagues [15] to calculate the energy cost of physical activities and the PAEE. This YCPA includes age-specific MET values for 196 activities for youth with all MET values derived from data of children and adolescents. Most previous validation studies have used either the adult compendium by Ainsworth and colleagues [29] or the old YCPA by Ridley and colleagues [30] to calculate energy expenditure. BMR per unit body mass in children is higher than in adults and gradually decreases with age. Hence, use of age-specific MET values can improve the accuracy of energy expenditure calculations [18] and this may have led to a higher correlation with less bias in estimating energy expenditure by the PAR-Q compared to the previously reported questionnaires [18–23].

Previous studies have deemed subjective physical activity measurement tools to be useful for estimating TEE at the population level for epidemiological research, if the percentage difference in means between TEE measured by DLW (TEE_DLW_) and TEE estimated by questionnaire or log (TEE_PAR-Q/PALog_) ((TEE_DLW_ - TEE_PAR-Q/PALog_)/TEE_DLW_) × 100% is less than 10%, and the correlation between these two estimates is greater than 0.60 [22, 31]. Accordingly, in the current study, PAEE calculated from the PAR-Q showed a better correlation value of 0.69 and physical activity log showed a correlation value of 0.49 with PAEE estimated by the criterion DLW technique, indicating PAR-Q as a better energy expenditure assessment method. However, the percentage difference in means for the PAR-Q and activity log were 10.3% and 0.7%, respectively.

There are many strengths in the current study, including the larger sample size compared to previous studies. Most similar validation studies that used the DLW technique had sample sizes less than 50. Cultural acceptability is another strength in the PAR-Q used in the current study. Most of the existing physical activity questionnaires are structured with a list of activities which may not capture all the different types and intensities of free-living physical activities that children and adolescents normally engage in. Validity results assessed in one population cannot be extrapolated to other populations with different ethnicity in other geographical regions [32]. The advantage of the PAR-Q used in the current study is that it allows participants to record the physical activities they perform facilitating its’ use in any country irrespective of cultural or ethnic differences in physical activities. Our observations support the use of PAR-Q in estimating PAEE in Sri Lankan adolescents.

## Conclusion

This is the first reported validation study of a physical activity recall questionnaire and activity log for Sri Lankan adolescents aged 11-13 years. The PAR-Q is a valid measure of physical activity among Sri Lankan adolescents at the group level compared to the physical activity log. The activity diary was less valid as there was a systematic bias with significantly underestimated energy expenditure at higher activity levels. Further PAR-Q provides information on intensity, frequency and duration of physical activities; it is an inexpensive and easy-to-use method for epidemiological studies.

## Data Availability

Researchers interested in accessing data are encouraged to contact the research team.
